# Minutes of the Ohio Dental College Association

**Published:** 1859-03

**Authors:** J. Taft

**Affiliations:** Secy.


### MINUTES OF THE OHIO DENTAL COLLEGE ASSOCIATION.

The association met according to adjournment, in the Dental College, Monday, 2 o'clock, Feb. 21st, 1859.

Present—Drs. J. Taylor, G. Watt, C. Bonsall, C. B. Chapman, J. Richardson, J. T. Toland, J. Taft, and fourteen others by proxy.

The meeting was called to order by the President, Dr. Chas. Bonsall, who made some very appropriate remarks, in regard to the condition of the institution, especially with reference to the session just closing.

Some general conference was held in regard to the condition of the institution.

Adjourned to meet at 3 o'clock, P. M., to-morrow.

### SECOND DAY.

Association met according to adjournment, at 3 o'clock, P. M., President in the chair. Minutes of the previous meeting were read and approved. The Treasurer presented his report, which after being audited by the committee, was accepted.

### *Report of the Treasurer.*

The Treasurer of the Ohio Dental College Association, would respectfully report that the floating debt of $251,40, as reported at our last annual meeting, stands now as follows:

| | |
|---|---|
| The amount due J. G. Douglas, at that time, has been reloaned by the same gentleman, and the association is in debt to the same, on 6th of March next, principal and interest | $282,65 |
| Amount due the Treasurer on bills paid up to the present time | 13,21 |
| Amout of floating debt | $295,86 |
| The amount in the Treasurer's hands at our last meeting, $47,30, to this $3 should be added, inaccurately footed up in our last report, making in the Treasurer's hands | $ 50,30 |
| Interest on the above, and deducting amount due the Treas. | 2,22 |
| Interest on $150 in the Treasurer's hands from 1st March to 1st July | 3,00 |
| | 55,52 |
| Deduct amount due Treasurer | 13,21 |
| | $ 42,31 |

Leaving forty-two dollars and thirty-one cents to be paid on note due Douglass.

| | |
|---|---|
| 1859, Jan. 1st, By cash of the Dean of the Faculty | $150,00 |
| Feb. 20th, " " " " " | 50,00 |
| | $200,00 |
| Interest of H. A. Smith | 6,00 |
| | $206,00 |
| Cr. Jan. 1st, Paid interest notes | 150,00 |
| Feb. 20th, " " " | 50,00 |
| | $200,00 |

The interest notes on the mortgages have been duly paid

JAS. TAYLOR, *Treasurer.*

Drs. W. W. Allport, of Chicago, Ill.; Edgar Taylor, Palmyra, Mo.; Eli Collins, Connersville, Ind.; B. B. Ward, Mobile, Ala.; D. W. Perkins, Milwaukee, Wis.; I. B. Branch, Galena, Ill.; J. M. Lewis, Marion, Ill., having become stockholders, were unanimously elected members of the association.

The Dean of the faculty read the following report:

### *Report of the Faculty.*

The Faculty of the Ohio Dental College, would respectfully submit the following report:

There has been no change in the Faculty during the last year, and we are happy to express the hope, that it will remain as at present.

Dr. H. A. Smith has filled the position as Demonstrator of Operative and Mechanical Dentistry, with commendable zeal, and to the entire satisfaction of all parties concerned; and we entertain the expectation that he will remain with us during the next session. The infirmary has been kept open during the past summer. The person placed in charge of it, remained only part of the summer; when he left, another was placed in charge. Throughout it was conducted to the entire satisfaction of those having control. The proceeds of the Infirmary were used for the improvement of the rooms connected with it. The Infirmary will probably not be kept open during the coming summer, owing to the pressing calls from abroad for the graduates. A preliminary course was commenced on the 18th of October, and the regular course on the first Monday of November.

We are happy to report an increase of the class over that of last session; numbering twenty-three. The character of the class is such that we are pleased to congratulate the institution therefor.

There are nine members of the graduating class, all of whom have complied perfectly with the requisitions of the institution.

*Names of Graduates.*—L. B. Hatch, Indiana; J. B. Doughtry, Mississippi; J. Ingalls, Wisconsin; J. B. Knisley, Ill.; G. B. Lenoir, Miss.; W. B. Ward, Ala.; E. Ruth, Ky.; O. Wilson, Ill.; S. Wardle, Ohio.

The following table embraces a list of the Infirmary operations during the sesion:

| | |
|---|---|
| Pivot Teeth | 10 |
| Pieces Repaired | 16 |
| Cases of Irregularity treated | 3 |
| No. of Gold fillings | 255 |
| Fillings of other material | 67 |
| Teeth Extracted | 368 |
| Cases of Salivary Calculus treated | 30 |
| Full Upper Sets of Continuous Gum Teeth | 3 |
| Full Upper Sets Specimens, " " | 2 |
| Partial Sets " " " | 3 |
| Full Set Cleoplasty, Practical | 1 |
| Under Sets " " | 2 |
| Under Sets Silver and Gold | 6 |
| Full Sets in Brass, Specimens | 8 |
| Full Upper Sets in Silver and Gold, practical | 9 |
| Partial Sets " " " | 8 |

| | |
|---|---|
| Full Set Block Work, Specimen | 1 |
| Partial Sets " Practical | 2 |
| Nerves destroyed | 10 |
| Fangs filled | 18 |
| Cases of Alveolar Abcess treated | 13 |

J. TAFT, *Dean.*

On motion, the association went into an election of officers, for the ensuing year.

| | |
|---|---|
| *President,* - | DR. CHAS. BONSALL. |
| *1st Vice President* | " W, R. WEBSTER. |
| *2d* " " | " W. W. ALLPORT. |
| *Secretary,* - | " J. TAFT. |
| *Treasurer,* - - | " J. TAYLOR. |

*Dental Examiners*—Drs. B. F. Maguire, New York City; J. F. Johnston, Indianapolis, Ind.; Dr. Dudley, Louisville, Ky.

*Medical Examiners*—Drs. B. F. Richardson, Cincinnati, Ohio; A. B. Butler, Richmond, Ind.

After some farther informal conference, the association adjourned to meet on Monday, 2 o'clock, P. M., Feb. 20 1860. J. TAFT, *Secy.*

